# Role of TLR5 and Flagella in *Bacillus* Intraocular Infection

**DOI:** 10.1371/journal.pone.0100543

**Published:** 2014-06-24

**Authors:** Salai Madhumathi Parkunan, Roger Astley, Michelle C. Callegan

**Affiliations:** 1 Departments of Microbiology and Immunology, University of Oklahoma Health Sciences Center, Oklahoma City, Oklahoma, United States of America; 2 Department of Ophthalmology, University of Oklahoma Health Sciences Center, Oklahoma City, Oklahoma, United States of America; 3 Dean A. McGee Eye Institute, Oklahoma City, Oklahoma, United States of America; Wayne State University, United States of America

## Abstract

*B. cereus* possesses flagella which allow the organism to migrate within the eye during a blinding form of intraocular infection called endophthalmitis. Because flagella is a ligand for Toll-like receptor 5 (TLR5), we hypothesized that TLR5 contributed to endophthalmitis pathogenesis. Endophthalmitis was induced in C57BL/6J and TLR5−/− mice by injecting 100 CFU of *B. cereus* into the mid-vitreous. Eyes were analyzed for intraocular bacterial growth, retinal function, and inflammation by published methods. Purified *B. cereus* flagellin was also injected into the mid-vitreous of wild type C57BL/6J mice and inflammation was analyzed. TLR5 activation by *B. cereus* flagellin was also analyzed *in vitro. B. cereus* grew rapidly and at similar rates in infected eyes of C57BL/6J and TLR5−/− mice. A significant loss in retinal function in both groups of mice was observed at 8 and 12 hours postinfection. Retinal architecture disruption and acute inflammation (neutrophil infiltration and proinflammatory cytokine concentrations) increased and were significant at 8 and 12 hours postinfection. Acute inflammation was comparable in TLR5−/− and C57BL/6J mice. Physiological concentrations of purified *B. cereus* flagellin caused significant inflammation in C57BL/6J mouse eyes, but not to the extent of that observed during active infection. Purified *B. cereus* flagellin was a weak agonist for TLR5 *in vitro*. These results demonstrated that the absence of TLR5 did not have a significant effect on the evolution of *B. cereus* endophthalmitis. This disparity may be due to sequence differences in important TLR5 binding domains in *B. cereus* flagellin or the lack of flagellin monomers in the eye to activate TLR5 during infection. Taken together, these results suggest a limited role for flagellin/TLR5 interactions in *B. cereus* endophthalmitis. Based on this and previous data, the importance of flagella in this disease lies in its contribution to the motility of the organism within the eye during infection.

## Introduction

*B. cereus* is a Gram-positive, sporulating bacterium that is more commonly recognized for causing food-borne illnesses, chronic skin infections, and systemic diseases such as meningitis and pneumonia [1]. Nosocomial infection pseudo-outbreaks caused by *B. cereus* have been reported in the last decade and have been attributed to contaminated disinfecting agents like ethyl alcohol [2] and alcohol swabs [3], or contaminated equipment like airflow sensors, intravenous catheters [1], [4], and ventilator and filtration units [1], [5], [6]. A recent nosocomial outbreak identified *B. cereus* in contaminated alcohol Prep Pads [7]. *B. cereus* is also highly associated with a blinding ocular infection termed endophthalmitis. Endophthalmitis is characterized by intraocular inflammation and damage to the retina, resulting in partial or complete loss of vision. Microbes can enter the posterior segment following an ocular injury (post-traumatic), surgery (post-operative) or from another site of infection (endogenous) [8], [9]. While cases of post-operative endophthalmitis generally respond positively to treatment, cases of post-traumatic and endogenous endophthalmitis caused by *B. cereus* have a significantly greater failure rate, necessitating the search for better strategies to combat the disease.

The pathogenicity of *B. cereus* in endophthalmitis is associated with the inflammogenicity of its cell wall and the production of secreted toxins and proteases [10]–[14]. Previous studies have shown that *B. cereus* endophthalmitis develops faster and is more virulent than endophthalmitis caused by other Gram-positive ocular pathogens such as *Staphylococcus aureus* [15], [16], *Enterococcus faecalis* [17], [18], or *Streptococcus pneumoniae* [19], [20]. The explosive nature of *B. cereus* endophthalmitis dictates the need for immediate and aggressive therapy to stop the progression of the disease. Currently, there is no universal therapeutic regimen which prevents vision loss that occurs during severe forms of endophthalmitis. The use of anti-inflammatory agents in addition to antibiotics has not proven effective [21]–[25]. In addition, current therapies ignore toxins which are proven to contribute to pathogen virulence in the eye [10], [12]–[19].

Innate immune mechanisms drive inflammation by the recognition of distinguishing molecules on the surface of the invading bacterium via a class of pattern recognition receptors called Toll-like receptors (TLRs) expressed on host cells. TLRs are expressed in ocular surface, retinal, iris, and corneal epithelial cells [26]–[28]. In the context of intraocular infections, TLRs have been found to be important in inflammation in *S. aureus* [29] and *B. cereus* [30] endophthalmitis. For experimental *B. cereus* endophthalmitis, the absence of TLR2 resulted in a diminished inflammatory environment when compared to controls [30], but there was still some degree of inflammation in *B. cereus*-infected TLR2−/− eyes. This suggests that other TLRs and/or components of innate immunity are involved in intraocular inflammation during *B. cereus* endophthalmitis.

When *B. cereus* infects the eye, the organism migrates rapidly throughout all parts of the eye, from the initial site of injection in the vitreous into the anterior segment within 6 to 12 hours [31]. This ability of *B. cereus* to migrate throughout the eye contributes to endophthalmitis pathogenesis [12], [32], [33]. The absence of motility affects toxin production and hence non-motile *Bacillus* caused less severe disease pathogenesis [12], [32], [33]. *B. cereus* use peritrichous flagella [34] as motility appendages which render the bacterium capable of movement throughout the eye. Moreover, flagella may impact the inflammatory response mounted against *B. cereus* since flagellin, the monomer which comprises full-length flagella, is a natural ligand for TLR5 [35]. Since *B. cereus* is a flagellated bacterium, we hypothesized that *B. cereus* flagella contributed to the pathogenesis during endophthalmitis by activating the ocular inflammatory response via TLR5. This hypothesis was tested by analyzing the immune response against *B. cereus* flagellin *in vitro* and *in vivo*, and by comparing the pathogenesis of *B. cereus* infection in an experimental model of endophthalmitis in wild type control and TLR5−/− mice.

## Methods

### Ethics Statement

These experiments involved the use of mice. All procedures were carried out in strict accordance with the recommendations in the Guide for Use of Laboratory Animals of the National Institutes of Health, institutional guidelines set forth by the University of Oklahoma Health Sciences Center IACUC, and the Association for Research in Vision and Ophthalmology Statement for the Use of Animals in Ophthalmic and Vision Research. The OUHSC IACUC approved these studies under protocols 11–068 and 11–090.

### Experimental *B. cereus* endophthalmitis

Wild type C57BL/6J mice were purchased from commercially available colonies (Stock No. 000664, Jackson Labs, Bar Harbor ME). An original breeding pair of TLR5−/− mice on the C57BL/6 background was a kind gift from Dr. Richard A. Flavell (Yale University, New Haven CT). Following rederivation, TLR5−/− mice were bred on the C57BL/6J background and maintained in-house on a 12 hour on/12 hour off light cycle under barrier facility conditions. All animals were acclimated to conventional housing after arrival/weaning for at least 2 weeks and were used in experiments at 8–10 weeks of age.

Experimental endophthalmitis was induced by injecting 100 CFU *B. cereus* strain ATCC 14579 into the mid-vitreous using a sterile capillary needle as previously described [30], [36]–[38]. At different time points postinfection, quantitation of intraocular bacterial growth, proinflammatory cytokines and chemokines, myeloperoxidase (MPO, to estimate PMN infiltration), and retinal function were performed, as described below.

### Intraocular Bacterial growth

Bacteria were quantified by harvesting infected eyes at 0, 4, 8, and 12 hours postinfection. The eyes were homogenized with 1 mm sterile glass beads (Biospec products, Inc., Bartlesville OK) in 400 µl PBS. Bacteria were then track diluted 10-fold onto brain-heart infusion (BHI) agar and quantified [30], [37], [38]. Values represent the mean ± standard deviation (SD) for N≥4 eyes per time point.

### Electroretinography

Retinal function was analyzed in wild type and TLR5−/− mice by electroretinography (ERG) as previously described [30], [37], [38]. ERGs were performed at 8 and 12 hours postinfection (Espion E2, Diagnosys LLC, Lowell MA). After dark adaptation for at least 6 hours, eyes were exposed to a transient flash of light. Bright flashes resulted in a response which consisted of an A wave initial negative amplitude followed by a B wave positive deflection. A-wave provides a direct measure of photoreceptor activity, while B-wave represents the action of Muller cells, bipolar cells, and second order neurons. A- and B-wave amplitudes were recorded for each infected eye and compared with the uninfected eye. The percentage of retinal function retained was then calculated using the formula 100 – {[1 – (experimental A-wave amplitude/control A-wave amplitude)] ×100} or 100 – {[1 – (experimental B-wave amplitude/control B-wave amplitude)] ×100} [38]. Values represent the mean ±SD for N≥4 eyes per time point.

### Histology

Whole eyes were harvested at 0, 4, 8, and 12 hours after infection and incubated in buffered zinc formalin fixative for 24 hours at room temperature [30], [37], [38]. Globes were then transferred to 70% ethanol and embedded in paraffin, sectioned, and stained with hematoxylin and eosin. Images are representative of 4 eyes/time point.

### Inflammatory Cell Influx

PMN influx into the eye was estimated by quantifying MPO levels in whole eye homogenates by sandwich ELISA (Mouse MPO ELISA Kit, Hycult Biotech, Plymouth Meeting PA), as previously described [30]. Eyes were harvested and analyzed for MPO activity at 0, 4, 8 and 12 h postinfection. Harvested eyes were suspended in PBS containing protease inhibitor cocktail (Roche Applied Science, Indianapolis IN) and homogenized. Homogenates of uninfected eyes served as negative controls. The lower limit of detection for this assay was 2 ng/ml. Results are reported as mean ±SD for N≥4 eyes per group per time point.

### Inflammatory Mediator Expression

Ocular proinflammatory cytokine and chemokine expression was quantified as previously described [30], [36], [37]. Eyes were harvested at 0, 4, 8 and 12 hours postinfection. Harvested eyes were suspended in PBS containing protease inhibitor cocktail and homogenized. Concentrations of IL6, TNFα, IL1β, and KC were quantified in harvested wild type and TLR5−/− eyes using commercial enzyme linked immunosorbent assay (ELISA) kits (Quantikine, R&D Systems, Minneapolis MN). The lower limits of detection for each assay were: TNFα, 2 pg/ml; KC, 2 pg/ml; IL6, 2 pg/ml; IL1β, 2 pg/ml. Values represent mean ±SD for N≥4 eyes/time point.

### Purification of *B. cereus* Flagellin

Flagellin preparations from *B. cereus* were generated based on a previously described method [39], [40]. Motile *B. cereus* was grown overnight in 1L Luria Bertani (LB) media with minimal rotary shaking (80 rpm) to avoid damage to intact flagella. Bacteria were harvested by centrifugation at 4000×g for 30 min, the pellet was resuspended in 15 ml of PBS containing protease inhibitor cocktail, and the suspension was vigorously mechanically shaken to remove the flagella. Two cycles of differential centrifugation were done at 15,000×g for 30 min to remove bacterial debris and again at 78,000×g for 2 h to sediment flagella. Purified flagella was then resuspended overnight in 1ml PBS with protease inhibitor and stored at 4°C. Purity of flagellar monomers was analyzed by SDS-PAGE. A single band of approximately 29 kD was identified, extracted from the gel, and sequenced (LC/MS/MS; OUHSC Laboratory for Molecular Biology and Cytometry Research, Oklahoma City OK). The purified flagellin protein sequence matched those of *B. cereus* ATCC 14579 flagellin (Accession No. gi|30019803) and *B. thuringiensis* flagellin A1 (Accession No. gi|189164115).

### Flagella-Induced Inflammation in the Eye

Purified *B. cereus* flagellin suspensions in PBS were injected into C57BL/6J or TLR5−/− mouse eyes as described above. 0.5 ng/0.5 µL flagellin were injected into each eye. ERGs were performed and eyes were harvested for histology at 0, 8, and 12 hours postinfection, as described above. ERG values represent the mean ±SD for N≥2 eyes/time point.

### Flagellin Activation of TLR5

Purified flagellin was tested for its ability to activate TLR5 in a TLR5 reporter cell line which expresses human TLR5 and secreted alkaline phosphatase reporter gene under the transcriptional control of NFκB (IML-105, TLR5/SEAPorter HEK 293 cells, Imgenex, San Diego CA). The positive control for this assay was purified flagellin FliC from *Salmonella typhimurium* (IMG-2205, Imgenex) tested at equal concentrations (0.1, 0.5, 1.0, 5.0, and 10.0 ng/mL). Results were analyzed by reading absorbance at 405 nm. Values represent mean ±SD for 2 replicates per concentration.

### Statistics

Results represent the arithmetic means ± standard deviations (SD) for all samples from each experimental group. A two-tailed, two-sample Student t test assuming equal variance was used to compare the statistical significance of the experimental groups. Statistical significance was determined at P≤0.05.

### Sequence Analysis

Flagellin sequences for *B. cereus* (NP_831435.1), *B. anthracis* (WP_001222388.1), *B. thuringiensis* (ABD33778.1), and *S. enterica* serovar *typhimurium* (S07276) were aligned, displayed, and analyzed with ClustalW (European Bioinformatics Institute, Cambridgeshire UK) [41].

## Results

### Intraocular Growth of *B. cereus*

**Figure 1:**
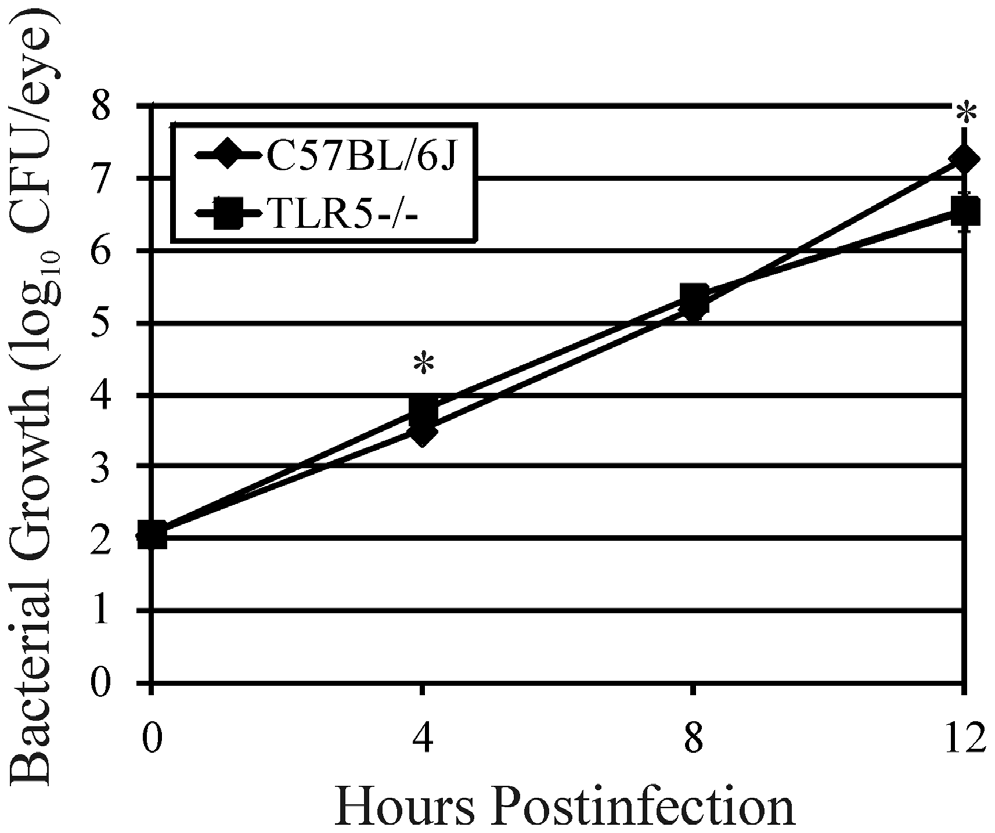
Influence of TLR5 on bacterial growth during experimental *B. cereus* endophthalmitis. C57BL/6J wild type and TLR5−/− mouse eyes were injected with 100 CFU *B. cereus*. Eyes were harvested, homogenized, and analyzed for bacterial growth. Overall, *B. cereus* grew to similar concentrations in infected eyes of TLR5−/− and C57BL/6J mice, suggesting that the absence of TLR5 did not influence the overall growth of *B. cereus* in the eye. Values represent the mean log_10_ CFU±SD of N≥4 eyes per time point for at least 2 separate experiments. *P≤0.05.

Bacterial growth in wild type C57BL/6J and TLR5−/− eyes is shown in Figure 1. The rates of bacterial growth followed a similar pattern but were statistically different at 4 hours (P = 0.002) and 12 hours (P = 0.01) postinfection. *B. cereus* reached maximum concentrations of approximately 6.5 (TLR5−/−) and 7.3 (C57BL/6J) log_10_ CFU/eye by 12 hours postinfection. This result suggested that the absence of TLR5 did not greatly affect the overall rate of *B. cereus* growth in the eye.

### Retinal Function

**Figure 2:**
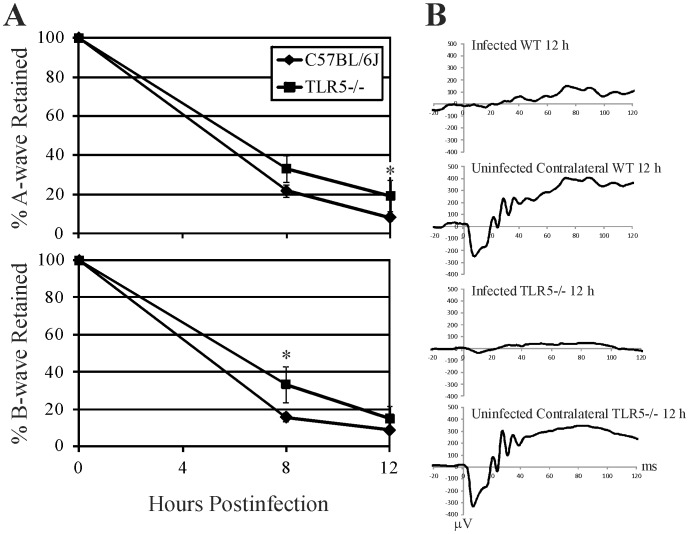
Influence of TLR5 on retinal function during experimental *B. cereus* endophthalmitis. C57BL/6J wild type and TLR5−/− mouse eyes were injected with 100 CFU *B. cereus*. Retinal function was assessed by electroretinography (ERG). **A**) A-wave amplitudes were slightly greater in C57BL/6J infected eyes at 12 h postinfection (P = 0.02), while B-wave amplitudes were greater in TLR5−/− infected eyes at 8 h postinfection (P = 0.008). By 12 hours postinfection, A-wave and B-wave amplitudes retained in both groups decreased to approximately 20% or less in infected eyes, indicating significant retinal function loss in both groups of mice regardless of the presence of TLR5. Values represent the mean ±SD of N≥4 eyes per time point for at least 2 separate experiments. *P≤0.05. **B**) Representative averaged waveforms from wild type (WT) and TLR5−/− mice at 12 h postinfection, with one eye infected and the contralateral eye serving as the uninfected control. Representative of N≥4 eyes per time point.

Retinal function analysis of *B. cereus* endophthalmitis in wild type C57BL/6J and TLR5−/− mice is summarized in Figure 2. Amplitudes of A-wave and B-wave declined significantly both in wild type C57BL/6J and TLR5−/− eyes at 8 and 12 hours following infection with *B. cereus*. The A-wave amplitudes in C57BL/6J infected eyes was similar at 8 h postinfection (P = 0.07), but slightly less at 12 h postinfection (P = 0.02). B-wave amplitudes retained were greater in TLR5−/− infected eyes at 8 h postinfection (P = 0.008), but both groups had similar ERG values at 12 h postinfection (P = 0.1). By 12 hours postinfection, both A-wave and B-wave amplitudes retained decreased to approximately 20% or less in infected eyes, indicating significant and comparable retinal function loss in both groups of mice.

### Intraocular Inflammation

**Figure 3:**
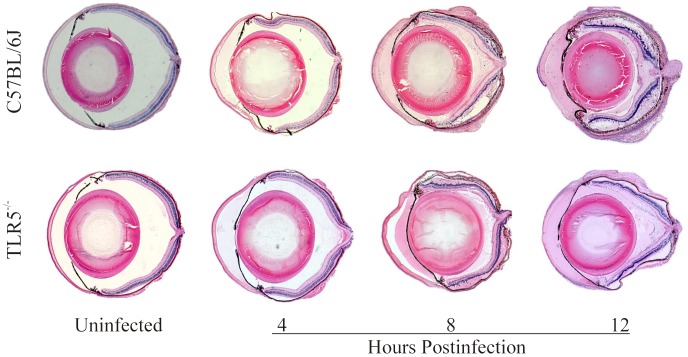
Whole eye histology of experimental *B. cereus* endophthalmitis. C57BL/6J and TLR5−/− mouse eyes were injected with 100 CFU *B. cereus*. Whole globes were harvested and processed for hematoxylin and eosin staining. Infected eyes of both groups had significant inflammation by 12 h postinfection, suggesting that the absence of TLR5 did not greatly impact intraocular inflammation. Sections are representative of 4 eyes per group. Magnification, 10X.

Histology of uninfected (control) and *B. cereus*-infected globes in wild type C57BL/6J and TLR5−/− mice is depicted in Figure 3. At 4 hours postinfection, wild type C57BL/6J and TLR5−/− mice had similar levels of fibrin deposition in the anterior segment and minimal fibrin and polymorphonuclear leukocyte (PMN) infiltration in the posterior segment. At this time, retinas were intact in eyes of both groups. At 8 hours postinfection, eyes of both groups had significant fibrin deposition in the anterior chamber and in the posterior segment, corneas were edematous, and significant numbers of PMN were present in the vitreous. In C57BL/6J and TLR5−/− mouse eyes, retinal layers were intact but retinal detachments were present. At 12 hour postinfection, whole globe inflammation was significant and retinal layers were indistinguishable in both groups of mice.

**Figure 4:**
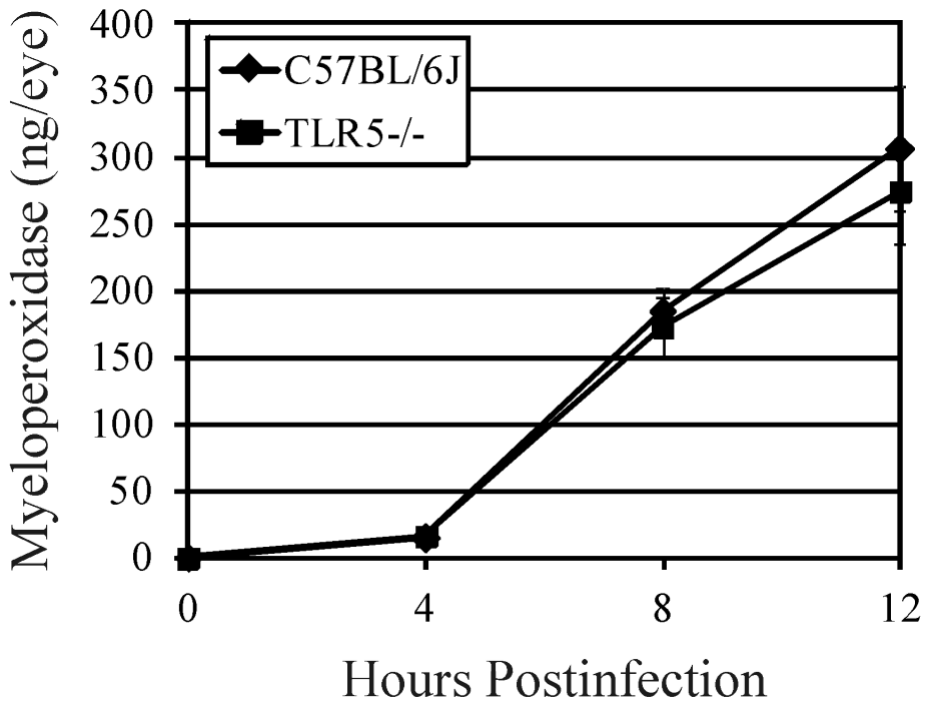
Influence of TLR5 on infiltration of PMN into mouse eyes during experimental *B. cereus* endophthalmitis. C57BL/6J and TLR5−/− mouse eyes were injected with 100 CFU *B. cereus*. PMN infiltration was estimated by quantifying MPO in whole eyes by sandwich ELISA. MPO levels were similar in these groups at all times points postinfection (P≥0.17), suggesting that the absence of TLR5 did not alter the PMN response during infection. Values represent the mean ±SD for N≥4 per group for at least 2 separate experiments. *P≤0.05.

PMN infiltration in whole eyes following *B. cereus* infection is depicted in Figure 4. Myeloperoxidase (MPO) levels increased significantly after 4 hours postinfection in C57BL/6J and TLR5−/− infected eyes. MPO levels were similar in these groups at all time points postinfection (P≥0.17). These results suggest that the absence of TLR5 did not alter the PMN response during infection, supporting the histology data.

**Figure 5:**
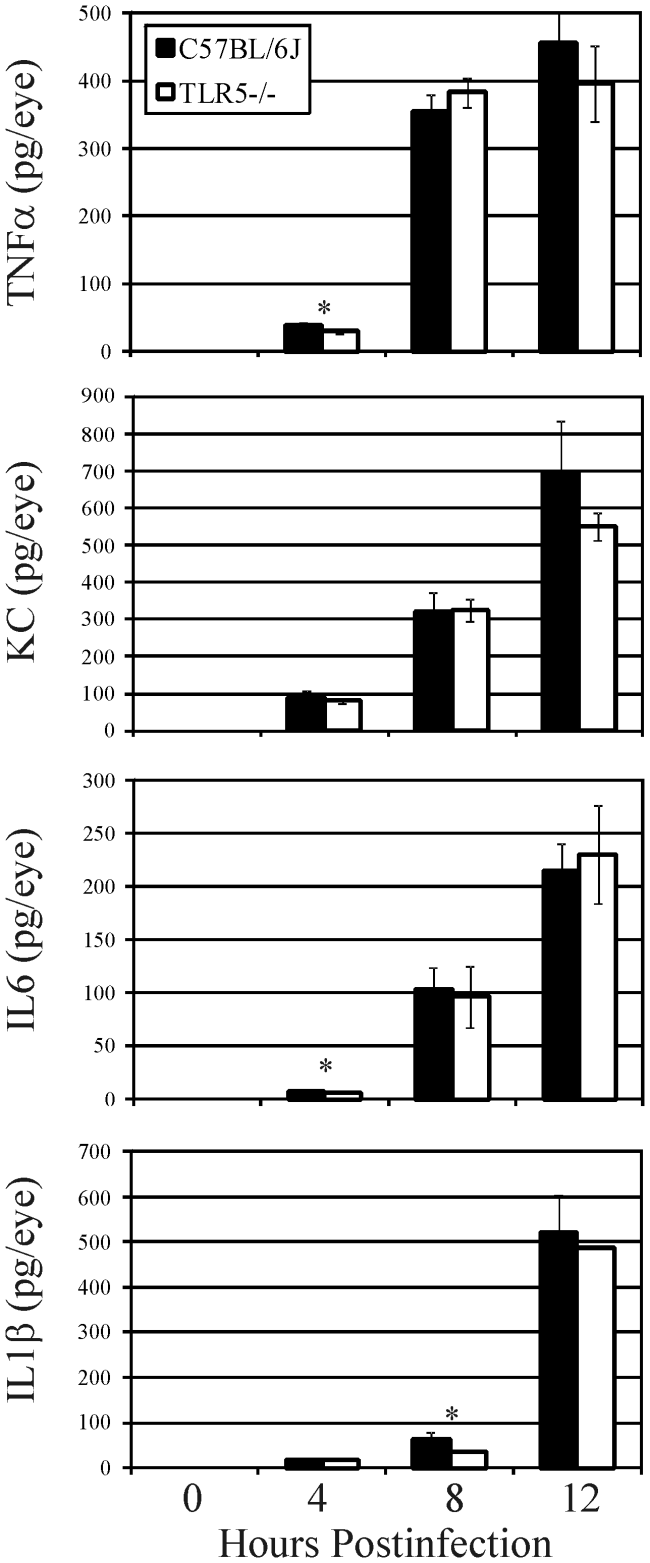
Influence of TLR5 on proinflammatory mediator expression during experimental *B. cereus* endophthalmitis. C57BL/6J and TLR5−/− mouse eyes were injected with 100 CFU *B. cereus*. Ocular proinflammatory cytokines and chemokines were analyzed by sandwich ELISA. Overall, similar levels of TNFα, KC, IL6, and IL1β were synthesized in infected eyes of C57BL/6J mice compared with that in infected eyes of TLR5−/− mice, suggesting that the absence of TLR5 did not alter the inflammatory mediator response during infection. Values represent the mean ±SD for N≥4 per group for at least 2 separate experiments. *P≤0.05.

The presence of proinflammatory cytokines and chemokines in the eye during infection is depicted in Figure 5. In general, all cytokines and chemokines tested increased significantly in both groups of mice during experimental endophthalmitis. TNFα levels were similar in C57BL/6J and TLR5−/− eyes at 8 and 12 hours postinfection (P≥0.1). TNFα levels increased approximately 12-fold in both groups between 4 and 12 hours postinfection. KC levels were similar at all time points postinfection (P≥0.05), with an approximate 7-fold increase in KC in both groups between 4 and 12 hours postinfection. IL6 levels were similar in C57BL/6J and TLR5−/− eyes at 8 and 12 hours postinfection (P≥0.57). IL6 levels increased an average of 30-fold in both groups between 4 and 12 hours postinfection. IL1β levels were similar in C57BL/6J and TLR5−/− mice, except at 8 hours postinfection when IL1β levels were slightly but significantly greater in infected C57BL/6J eyes (P = 0.001). IL1β levels increased 8-fold and 13-fold in C57BL/6J and TLR5−/− mouse eyes between 8 and 12 hours postinfection (P≤ 0.0001). Despite a few time points where proinflammatory mediators were slightly but significantly greater in C57BL/6J eyes compared to that of TLR5−/− eyes, the data suggest that overall, the cytokine and chemokine response to *B. cereus* infection was not altered by the absence of TLR5. These results coincided with the histology and MPO data, indicating that TLR5 did not significantly contribute to the inflammatory response in experimental *B.cereus* endophthalmitis.

### Intraocular Effects of Flagellin

**Figure 6:**
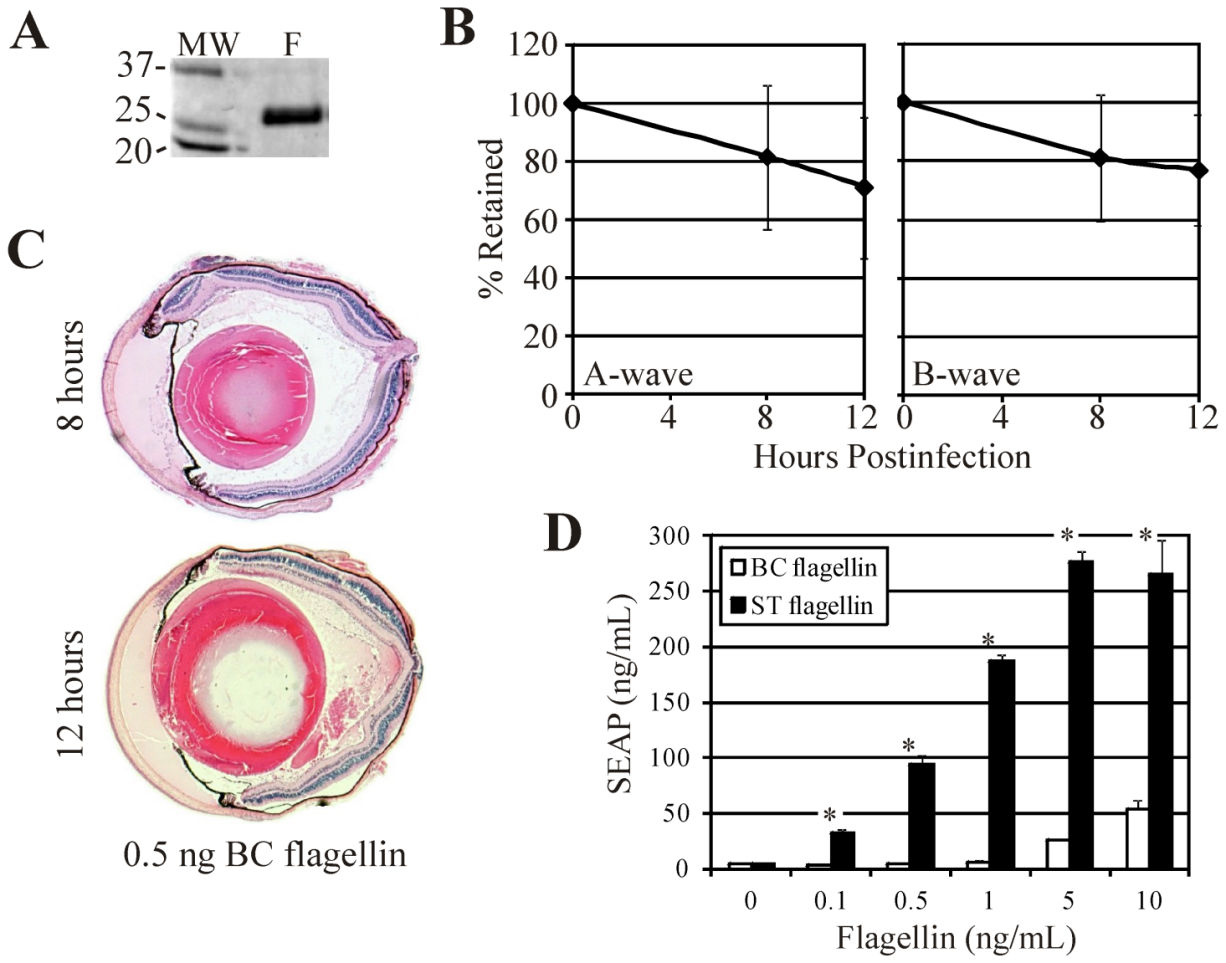
Role of flagellin in intraocular inflammation. A) Purified *B. cereus* flagellin was injected into C57BL/6J mouse eyes. *B. cereus* flagellin was purified as described in the Methods and analyzed for purity on a SDS-PAGE/Coomassie gel. A single band of 29 kD was recovered (lane F). **B and C**) Following injection of 0.5 ng/0.5 µL of purified flagellin, eyes were analyzed by ERG and whole globe histology at 8 and 12 hours. At 8 hours, posterior segment inflammation was minimal, retinas were intact, and retinal function decreased slightly (but not significantly) from that at time 0 (Figure 6B, P≥0.4). Compared with the significant inflammation and retinal function loss observed during infection, these results suggest that *B. cereus* flagellin alone may not have contributed significantly to the process. Infected eyes at 12 hours (Figure 3) demonstrated much greater pathology than that seen at 12 hours in eyes injected with flagellin alone. **D**) TLR5 activation by purified *B. cereus* (BC) flagellin was compared with that of purified *Salmonella typhimurium* (ST) flagellin. *B. cereus* flagellin was a weak TLR5 agonist, resulting in significantly less NFκB activity at comparable flagellin concentrations (mean ±SD for two repeated experiments, *P≤0.001).

It has been reported that flagellin monomers elicit a TLR5-mediated response because the flagellar TLR5-binding domain is exposed in monomers, but not in polymerized flagellin [42], [43]. These findings were reported for *Salmonella* and *Pseudomonas* flagellin, but, to our knowledge, these effects have not been analyzed for *B. cereus* flagellin. To determine whether flagellin alone could cause intraocular inflammation in the absence of *B. cereus* organisms, flagellin monomers (Figure 6A) were purified and intravitreally injected into mouse eyes as described in the Methods. Based on a report of an average of 11 flagella per *B. cereus* cell [44], an estimated 20,000 flagellin subunits per filament [45], and a calculated molecular mass of 29 kD [46], we estimated that injecting 0.5 ng flagellin into an eye would equate to that quantity of flagellin found in 4.72×10^5^ CFU *B. cereus*. Extrapolation of the CFU data in Figure 1 suggests that this concentration of *B. cereus* was present in the mouse eye at approximately 8 hours postinfection, a time when retinal function loss and inflammation were significant in infected eyes (Figures 2–4). At 8 hours following injection of 0.5 ng of purified flagellin, posterior segment inflammation was minimal and retinal function decreased slightly (but not significantly) from that at time 0 (Figure 6BC, P≥0.4). These results suggest that *B. cereus* flagellin alone may not have contributed significantly to retinal function loss or inflammation during an actual infection. Delayed intraocular inflammation was observed at 12 h following injection of 0.5 ng flagellin (Figure 6C); however, infected eyes at 12 h (Figure 3) demonstrated much greater pathology than that seen at 12 hours in eyes injected with flagellin alone. No posterior segment inflammation was observed in eyes injected with 100-fold less purified flagellin (data not shown). Injection of 0.5 ng flagellin into TLR5−/− eyes resulted in slightly less but still significant inflammation and similar retained A-wave (P = 0.16) and B-wave (P = 0.76) amplitudes compared to that of wild type eyes at 12 h postinjection (Figure S1). These results suggest that TLR5 may not be essential to intraocular inflammation caused by flagellin, and that flagellin, when present, may induce inflammation through a different pathway when TLR5 is absent.

Because the degrees and timing of intraocular inflammation present in infected eyes versus those injected with purified flagellin differed so greatly, we analyzed whether *B. cereus* flagellin was an agonist of TLR5. TLR5 activation of purified *B. cereus* flagellin was compared with that of purified *Salmonella typhimurium* flagellin *in vitro*. Compared to *Salmonella* flagellin, *B. cereus* flagellin was a weak TLR5 agonist, resulting in significantly less NFκB activity at comparable flagellin concentrations (Figure 6D). Taken together, these results suggest that *B. cereus* flagellin/TLR5 interactions in the eye may not be significant enough to greatly impact the overall course of intraocular inflammation during experimental endophthalmitis.

## Discussion

TLR5 is an important innate immune regulator of inflammation in infections, including those caused by *Salmonella* [47], [48], *Legionella* [49], [50], *Clostridium* [51], *Pseudomonas* [52], [53], *E. coli* [54], and others. TLR5 is also an important mediator of gut homeostasis [55]. TLR5 has been detected in cells of the eye [27], [56], [57]. TLR5 has been reported to modulate corneal inflammation and the innate antimicrobial response *in vitro* [58]–[60] and is important in ocular inflammation during bacterial and fungal keratitis [52], [61]. We therefore sought to determine the role of TLR5 in endophthalmitis caused by *B. cereus*, an organism which possesses the TLR5 ligand, flagella.

The majority of studies on flagellin/TLR5 interactions have been done with Gram-negative organisms. Few studies have analyzed interactions between Gram-positive flagella and TLR5. *Listeria* and *Clostridium* flagellin have been shown to be TLR5 agonists [35], [62]. Although *B. subtilis* flagellin is commercially marketed as a TLR5 agonist, *in vitro* results disagree on its ability to activate TLR5 [56], [63]–[65]. The *B. cereus sensu lato* group, including pathogens *B. cereus*, *B. anthracis*, and *B. thuringiensis*, have peritrichous flagella. Because the motility of *B. cereus*, and therefore its flagella, are important in the virulence of *B. cereus* during endophthalmitis [8], [66], we hypothesized that flagellin/TLR5 interactions also contributed to the pathogenicity of infection.

Despite our finding that *B. cereus* flagellin was a weak TLR5 agonist *in vitro*, physiological concentrations of purified B. cereus flagellin monomers caused inflammation in wild type and TLR5−/− mouse eyes. However, this inflammation was dissimilar to the degree of inflammation caused by active infection when that concentration of flagellin would have been present in the eye. *B. cereus* migrates through all parts of the eye during endophthalmitis [31], so its flagella are likely polymerized. The disparity could therefore be explained by the fact that flagellin monomers of other organisms, but not polymerized flagellin, have been shown to activate TLR5 [42], [43]. If flagellin monomers were not present in the eye during infection, this may explain why the absence of TLR5 did not significantly impact intraocular inflammation during infection. The results also suggest that unlike the environment in the inflamed gut where high levels of flagellin monomers exist [67], [68], flagellin monomers are either not present or are present at non-inflammogenic concentrations in the eye during endophthalmitis.

The potential lack of a significant role for polymerized flagellin in intraocular inflammation also brings forth an interesting question about the physiological state of *B. cereus* in the eye during infection. We demonstrated that mutant *B. cereus* which cannot swarm do not migrate into the anterior segment and cause a less virulent infection than wild type *B. cereus* that can swarm [33]. *In vitro*, swarming cells are elongated and hyperflagellated on media [69], but the swarming state of *B. cereus* in the eye during endophthalmitis has not been analyzed. If *B. cereus* is in a physiological state of swarming in the eye, then our concentration of flagellin injected into the eye may have been too low, as 40-fold increases in flagellin have been reported for swarming *B. cereus* [70]. However, if flagellin monomers were not present in the eye during infection, the increased number of flagella present in swarming organisms would be irrelevant, and TLR5/flagellin interactions would still not be as important to the outcome of infection.

**Figure 7:**
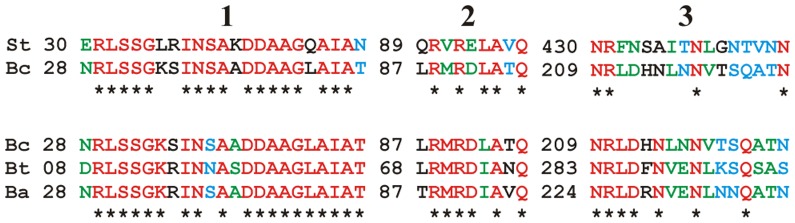
Multiple sequence alignments of *S. typhimurium*, *B. cereus*, *B. thuringiensis*, and *B. anthracis* flagellins. The amino acid sequences of *B. cereus* (Bc) and *S. typhimurium* (St) (top) and *Bc, B. thuringiensis* (Bt), and *B. anthracis* (Ba) (bottom) were aligned by ClustalW [71], focusing on regions important for IL8 activity (Region 1) and TLR5 stimulation and recognition (Regions 2 and 3). Asterisks and red letters identify amino acids conserved between Bc and St sequences or among Bc/Ba/Bt sequences. Blue amino acids have strongly similar properties, while green amino acids have weakly similar properties. Bc and St had 81% conserved residues in Region 1, 62.5% conserved residues in Region 2, and 25% conserved residues in Region 3. Bc, Ba, and Bt had 86% conserved residues in Region 1, 62.5% conserved residues in Region 2, and 44% conserved residues in Region 3.

*B. cereus* and *B. subtilis* do not fall into the category of organisms whose flagellin is not recognized by TLR5 [71]. Therefore, a TLR5 evasion mechanism similar to that demonstrated by *Helicobacter* or *Campylobacter* [71] may not be occurring here. ClustalW alignments of the *B. cereus* ATCC 14579 and *S. typhimurium* H1-A flagellin sequences demonstrated significant similarity (81%) in a region shown to be important for IL8 activity in Caco2 cells (amino acids 30−52) (Figure 7) [72]. However, ClustalW comparisons of these flagellin sequences also demonstrated that *B. cereus* flagellin contains differences in the TLR5 recognition and binding sites. The *S. typhimurium* FliC-TLR5 stimulatory activity lies within amino acids 89-96 in the N-terminal D1 domain [43]. Important residues for TLR5 activation also exist in the C-terminal conserved domain (430–445) [43]. Additional residues located between the IL8 activity region and the N-terminal D1 domain (58, 59 of *S. typhimurium*) and within the C-terminal D1 domain (411 of *S. typhimurium*) are also required for TLR5 recognition, as these residues are in physical contact with the N-terminal TLR5 binding region [43]. *B. cereus* shares 62.5% identity with the 89-96 region of *S. typhimurium*, including identity with three amino acids deemed essential for TLR5 binding activity, protofilament assembly, and motility [43]. Only 25% of the residues in the C-terminal conserved domain are identical between these flagellins. Residues 58, 59, and 411 were not identical, suggesting that the three dimensional structure of TLR5 binding by *B. cereus* flagellin is different from that of *S. typhimurium*. This is not a surprise, as *B. cereus* flagellin is 221 residues shorter than *S. typhimurium* FliC. Whether or not these differences account for the reduced TLR5 agonism of *B. cereus* flagellin or whether this lack of agonism extends to other members of the *B. cereus sensu lato* group (Figure 7) is an open question. A recent report supports the idea of differential activation of TLR5 and NAIP5/NLRC4 inflammasome receptors by the flagellins of different organisms [73]. In evaluating the use of *Bacillus cereus sensu lato* group flagellins for vaccine development, species-specific differences in these domains are important to consider.

Our results demonstrated that *B. cereus* flagella/TLR5 interactions, if present, did not contribute significantly to endophthalmitis pathogenesis. Although *B. cereus* flagella may not have contributed to inflammation during infection, its role in migration throughout the eye during infection is clearly important. We previously demonstrated that non-motile and non-swarming flagellated mutants are significantly less virulent than their motile and swarming wild type parental strains [32], [33]. Therefore, immobilization of the organism is paramount during the early stages of infection. Realistically, this would be achieved with appropriate administration of bactericidal antibiotics at the site of infection as early as possible during the infection course to sterilize the eye [25]. However, antibiotics do not inactivate the multitude of toxins synthesized by *B. cereus* or other organisms in the eye during infection which contribute to intraocular virulence. Future efforts to improve the visual outcome of patients with endophthalmitis caused by *B. cereus* and other virulent pathogens should include anti-toxin strategies with sterilization and better anti-inflammatory drugs to prevent the inflammation and tissue damage which results in vision loss during this disease.

## Supporting Information

Figure S1 **Flagellin causes similar inflammation and retinal function changes in wild type and TLR5−/− mice.** **A**) Purified *B. cereus* flagellin (0.5 ng) was injected into C57BL/6J mouse eyes as depicted in Figure 6. Injection of flagellin resulted in slightly less but still significant inflammation in TLR5−/− eyes compared to that of wild type eyes (representative of N = 3 TLR5−/− eyes at 12 h postinjection. **B**) Eyes underwent electroretinography as depicted in Figure 6. At 12 h postinjection, retained A-wave (P = 0.16) and B-wave (P = 0.76) amplitudes were similar between wild type and TLR5−/− eyes (mean ±SD, N≥2/group).
(TIF)
