# Supplementary figures and images for: Role of TLR5 and Flagella in Bacillus Intraocular Infection
Source: PLoS One. 2014 Jun 24;9(6):e100543. doi: 10.1371/journal.pone.0100543 (PMC4068998; doi:10.1371/journal.pone.0100543)

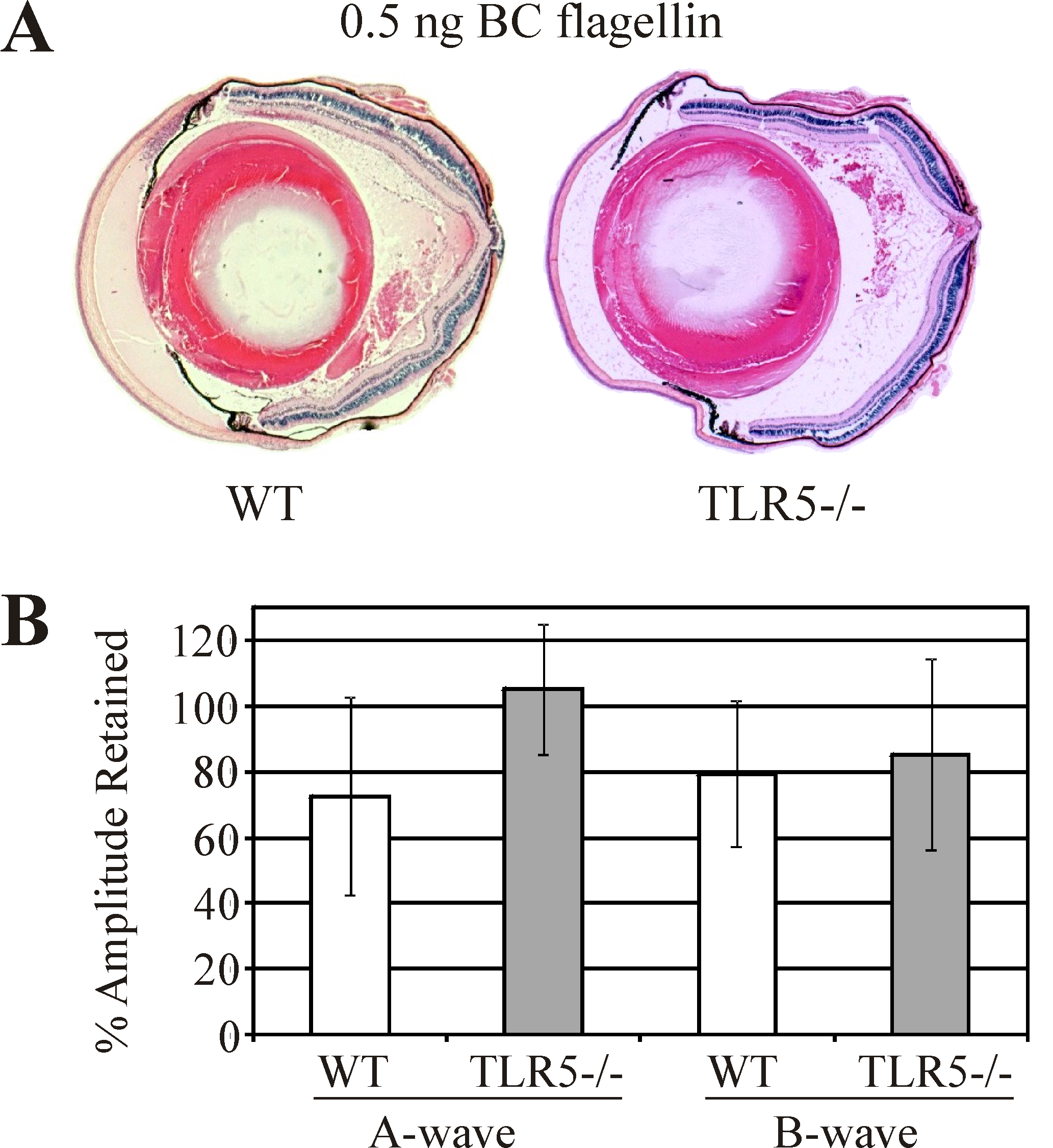

Supplement: Figure S1 — Flagellin causes similar inflammation and retinal function changes in wild type and TLR5−/− mice. A) Purified B. cereus flagellin (0.5 ng) was injected into C57BL/6J mouse eyes as depicted in Figure 6. Injection of flagellin resulted in slightly less but still significant inflammation in TLR5−/− eyes compared to that of wild type eyes (representative of N = 3 TLR5−/− eyes at 12 h postinjection. B) Eyes underwent electroretinography as depicted in Figure 6. At 12 h postinjection, retained A-wave (P = 0.16) and B-wave (P = 0.76) amplitudes were similar between wild type and TLR5−/− eyes (mean ±SD, N≥2/group). (TIF) [file pone.0100543.s001.tif]
